# Zika virus infection damages the testes in mice

**DOI:** 10.1038/nature20556

**Published:** 2016-10-31

**Authors:** Jennifer Govero, Prabagaran Esakky, Suzanne M. Scheaffer, Estefania Fernandez, Andrea Drury, Derek J. Platt, Matthew J. Gorman, Justin M. Richner, Elizabeth A. Caine, Vanessa Salazar, Kelle H. Moley, Michael S. Diamond

**Affiliations:** 1 Departments of Medicine, The Center for Human Immunology, Washington University School of Medicine, Saint Louis, MO 63110 USA; 2 Departments of Obstetrics and Gynecology, The Center for Human Immunology, Washington University School of Medicine, Saint Louis, MO 63110 USA; 3 Departments of Pathology and Immunology, The Center for Human Immunology, Washington University School of Medicine, Saint Louis, MO 63110 USA; 4 Departments of Molecular Microbiology, The Center for Human Immunology, Washington University School of Medicine, Saint Louis, MO 63110 USA; 5 Departments of Cell Biology and Physiology, The Center for Human Immunology, Washington University School of Medicine, Saint Louis, MO 63110 USA; 6 Departments of Immunotherapy Programs, Washington University School of Medicine, Saint Louis, MO 63110 USA

## Abstract

Zika virus (ZIKV) infection of pregnant women can cause congenital malformations including microcephaly, which has focused global attention on this emerging pathogen^1^. In addition to transmission by mosquitoes, ZIKV can be detected in the seminal fluid of affected males for extended periods of time and transmitted sexually^2^. Here, using a mouse-adapted African ZIKV strain (Dakar 41519) we evaluated the consequences of infection in the male reproductive tract of mice. We observed persistence of ZIKV, but not the closely related Dengue virus (DENV), in the testis and epididymis of male mice, and this was associated with tissue injury that caused diminished testosterone and inhibin B levels, and oligospermia. ZIKV preferentially infected spermatogonia, primary spermatocytes, and Sertoli cells in the testis, resulting in cell death and destruction of the seminiferous tubules. Less damage was observed with a contemporary Asian ZIKV strain (H/PF/2013), in part because this virus replicates less efficiently in mice. The extent to which these observations in mice translate to humans remains unclear, but longitudinal studies of sperm function and viability in ZIKV-infected humans seem warranted.

We and others have observed that ZIKV infection of male adult mice results in infection of the testes^3,4^, which is consistent with observed male-to-female^5,6^ and male-to-male^7^ sexual transmission in humans. To address the consequences of infection on the male reproductive tract, we performed a longitudinal study in wild-type (WT) C57BL/6 mice infected with ZIKV (strains H/PF/2013 (French Polynesia 2013) or mouse-adapted Dakar 41519 (Senegal 1984)) or DENV serotype 2, strain D2S20). Because ZIKV and DENV do not efficiently antagonize type I IFN signaling in mice compared to humans^8^, animals were treated with a single dose of anti-Ifnar1 blocking monoclonal antibody to facilitate infection and dissemination. When WT mice were treated instead with an isotype control antibody and then infected, ZIKV RNA did not accumulate in the testes (Fig 1a).

In the presence of anti-Ifnar1 antibody, high levels of viral RNA (10^5^ to 10^8^ focus-forming unit (FFU) equivalents/g or ml) and infectious virus (up to 10^8^ plaque forming units (PFU)/g or ml) were detected in the testis, epididymis, and the fluid collected from the epididymis within seven days of infection with either of the two ZIKV strains but not DENV (Fig 1a–c). ZIKV-Dakar replicated to higher levels compared to ZIKV-French Polynesia, which is consistent with its enhanced virulence in WT mice^3^. Remarkably, ZIKV RNA and infectious virus also were detected in mature sperm harvested from the epididymis (Fig 1b–c, and Extended Data Fig 1). At day 7 after inoculation, ZIKV-infected testes appeared similar in size to uninfected testes from age-matched mice and had equivalent weights (Fig 1d–e). Histological analysis of ZIKV-infected testis and epididymis at day 7 revealed no apparent differences in architecture (Fig 1f and Extended Data Fig 2). However, staining for CD45 (a pan-leukocyte marker) was observed in testis sections only from ZIKV-infected animals, with CD45^+^ cells localizing to the interstitium between the seminiferous tubules (Fig 1g, *column 1*). The blood-testis-barrier (BTB) remained intact at day 7 after infection, as judged by equivalent staining of the ETV5 transcription factor (which mediates BTB function and testicular immune privilege^9^) in Sertoli and germ cells in sections from uninfected and ZIKV-infected mice (Fig 1g, *column 2*). Furthermore, there was no CD45 staining on the seminiferous tubular side of the BTB, near the TRA98^+^ germ cells or spermatogonia (Fig 1g, *column 1*). A similar pattern of CD45 staining in the testicular interstitium and epididymal epithelium was described in patients infected with HIV^10^; indeed, we also observed scattered CD45^+^ cells in the epididymal epithelium of ZIKV-infected mice (Fig 1g, *column 5*). However, at day 7, F4/80^+^ macrophages were not apparent in the testicular interstitium or the lumenal epithelium of the epididymis of ZIKV-infected mice (Fig 1g, *columns 3 and 4*).

To determine which cells were targeted by ZIKV, we performed *in situ* hybridization (ISH) for viral RNA at day 7 after infection. In the testis, ZIKV RNA was evident in spermatogonia, primary spermatocytes, and the trophic, inhibin B-producing Sertoli cells (Fig 1h, *left panels*), with relative sparing of the androgen-producing Leydig cells. In the cauda epididymis, mature sperm in the lumen stained prominently for ZIKV RNA (Fig 1h, *right panels*) as did sperm cells liberated from the epididymis (Extended Data Fig 1).

We followed the consequences of ZIKV infection of the male reproductive tract over time. At day 14 after inoculation, high levels of ZIKV RNA persisted in the testis, epididymis, the fluid from the epididymis, and mature sperm of most mice (Fig 2a). In ZIKV Dakar-infected animals, there was a noticeable decrease in testis size and weight (Fig 2b–c). In comparison, no appreciable infection by DENV was observed in the testis at this time point (Extended Data Fig 3a). Histological analysis of the ZIKV-infected testis at day 14 showed damage to the architecture of the seminiferous tubules with a loss of the central ductal lumen (Fig 2d). This was associated with decreased numbers of TRA98^+^ germ cells, Lina28a^+^ type A and B spermatogonia, morphologically abnormal GATA4^+^ Sertoli cells, and detachment of Sertoli cells from the basement membrane (Fig 2e and Extended Data Fig 2). In some regions, large numbers of CD45^+^ leukocytes were observed, suggesting a substantive inflammatory cell infiltration (Fig 2d, *left panels;* Fig 2e, *column 1*). The absence of ETV5^+^ cells at this time point indicates loss of integrity of the BTB, which could explain the extent of interstitial inflammation and F4/80^+^ macrophages in the affected testis. The epididymis of ZIKV-infected animals also showed tissue injury at day 14, as reflected by constriction of the epididymal lumen, thickening of inter-luminal tissue, and accumulation of sperm interspersed with necrotic bodies (Fig 2d–e, *right panels*). ISH at day 14 showed progressive evidence of ZIKV RNA in cells of the testis, in the mature lumenal sperm, and on cilia layering the inner lumen of the epididymis, similar to day 7 (Fig 2f).

High levels of viral RNA persisted in tissues of the male reproductive tract at 21 days after ZIKV-Dakar inoculation (Fig 3a), and this was associated with loss of tissue architecture. Involution of the testis was observed as reflected by their noticeably reduced size and weight (Fig 3b–c). Histological analysis revealed almost complete destruction of the seminiferous epithelium with constricted tubules after ZIKV infection (Fig 3d). The populations of spermatogonia, Sertoli cells, and 3β-HSD^+^ Leydig cells were markedly diminished, and this was associated with persistent CD45^+^ leukocyte infiltration (Fig 3e and Extended Data Fig 2). In the epididymis, ZIKV infection resulted in constriction of the lumen with a mass of residual sperm that was interspersed with necrotic bodies (Fig 3d). ISH showed viral RNA in remaining testicular cells of the damaged testes and in the lumenal sperm of the infected epididymis (Fig 3f). Damage to the seminiferous tubules in the testis, albeit at lower levels, also was observed with the epidemic ZIKV H/PF/2013 strain at day 28 after infection (Extended Data Fig 3b–c).

The RNA ISH analysis suggested that Sertoli cells were targeted by ZIKV in the testes. Sertoli cells provide a trophic function for spermatogenesis and express high levels of TAM receptors Tyro3, Axl, and Mertk^11^. As Axl recently has been postulated as an entry factor for ZIKV infection of cells^12-16^, we assessed the impact of a genetic deficiency of Axl on ZIKV infection of the testis and epididymis. As we found high levels of infection in the testis and epididymis in *Axl*^-/-^ mice (Extended Data Fig 4a), this TAM receptor likely does not have an essential role in ZIKV pathogenesis in the male reproductive tract. ISH revealed prominent staining of viral RNA in both Sertoli and germ cells in *Axl*^-/-^ mice at day 7 after ZIKV infection (Extended Data Fig 4b).

The histological analysis revealed injury of the testis was associated with inflammatory cell infiltration. To assess the role of adaptive immune cells in the pathogenesis of acute disease, we inoculated *Rag1*^-/-^ mice, which lack both mature B and T cells, with ZIKV after a similar treatment with anti-Ifnar1 antibody. At day 7, we observed high levels of viral RNA in all male reproductive tract tissues (Extended Data Fig 4a). At day 13, we observed ZIKV RNA in germ and Sertoli cells in *Rag1*^-/-^ mice, and this was associated with fewer TRA98^+^ germ cells and Lin28a^+^ spermatogonia and breakdown of the BTB. However, interstitial Leydig cells remained in ZIKV-infected *Rag1*^-/-^ mice even though the architecture of the seminiferous tubules was altered (Extended Data Fig 4c–d). Thus, damage to the testis appears mediated both by ZIKV infection and adaptive immune responses.

To determine the functional consequences of ZIKV-Dakar infection in the testis, we measured the levels of two hormones important for spermatogenesis, testosterone and inhibin B, which are produced by Leydig and Sertoli cells, respectively. At day 7 after ZIKV infection, testosterone levels in homogenates of testes were higher, possibly because of the altered cellular physiology or inflammatory environment associated with viral replication^17^. By day 14, testosterone levels in ZIKV-infected mice were decreased and remained low at 21 days (Fig 4a, *left panel*). Inhibin B levels also were lower in ZIKV-infected testes at days 14 and 21 after infection (Fig 4a, *right panel*). We observed diminished total and motile sperm counts from fluid harvested from the cauda epididymis at days 14 (Fig 4b) or ∼42 (Fig 4c) days after ZIKV inoculation, which was consistent with extensive damage to the seminiferous tubules (Fig 2d–f, Extended Data Fig 2 and 5a–b). We also observed reduced rates of pregnancy and number of viable fetuses from females mated with ZIKV-infected compared to uninfected males (Fig 4d). Consistent with significant injury to the testis, there was marked cell death in the seminiferous tubules and lumen of the epididymis at multiple time points, as judged by TUNEL staining (Fig 4e) and loss of cellularity (Fig 3e–g). Thus, in mice, the injury to the male reproductive tract due to ZIKV infection results in decreased sex hormone production and oligospermia. ZIKV pathogenesis in the testis appears distinct from mumps virus, which preferentially infects interstitial Leydig cells and causes a highly inflammatory acute orchitis^18,19^.

In most human infections, ZIKV causes a mild febrile illness associated with rash and conjunctivitis. However, severe phenotypes now are appreciated including Guillain-Barré syndrome^20,21^ and congenital abnormalities in fetuses^22^. In contrast to related flaviviruses, ZIKV can be transmitted sexually, as infectious virus persists in the semen of males^23-25^ for up to 80 days after symptom onset^2^. Our experiments with mouse-adapted ZIKV Dakar show that infection causes testicular and epididymal damage in mice that can progress to reduction in key sex hormones, destruction of germ and somatic cells in the testis, and loss of mature sperm and fertility. Sertoli cells may be a key target for ZIKV in the testis, resulting in cell dysfunction, detachment from the basement membrane, and dissolution of the BTB. Infiltrating inflammatory cells may amplify destruction of the testicular architecture. Although further studies are required, this pathologic process results in decreased male fertility, at least in mice. While Axl is not required for infection of the mouse testis, other TAM or TIM^15^ receptors could be important for ZIKV tropism.

The establishment of a model of male reproductive tract injury after ZIKV infection will allow the rapid testing of new classes of therapeutic agents^26,27^ or vaccines^28^ to mitigate or prevent disease. Although our data are concerning for yet another unanticipated clinical manifestation of ZIKV infection, we acknowledge these results reflect studies exclusively performed in mice. Nonetheless, genitourinary signs and symptoms in ZIKV-infected humans have been reported including hematospermia, dysuria, and perineal pain^5,6,29^, and ZIKV recently was detected in human spermatozoa^30^. Longitudinal studies monitoring ZIKV infection in semen and sperm counts seem warranted to define the extent and consequences of this disease process in affected human males.

## Methods

### Ethics statement

This study was carried out in accordance with the recommendations in the Guide for the Care and Use of Laboratory Animals of the National Institutes of Health. The protocols were approved by the Institutional Animal Care and Use Committee at the Washington University School of Medicine (Assurance number A3381-01). Inoculations were performed under anesthesia induced and maintained with ketamine hydrochloride and xylazine, and all efforts were made to minimize animal suffering.

### Viruses

ZIKV strain H/PF/2013 (French Polynesia, 2013) was provided by the Arbovirus Branch of the Centers for Disease Control and Prevention with permission (X. de Lamballerie, Aix Marseille Université). ZIKV strain Dakar 41519 (Senegal, 1984) was provided by the World Reference Center for Emerging Viruses and Arboviruses (R. Tesh, University of Texas Medical Branch) and passaged twice in *Rag1*^-/-^ mice to create a mouse-adapted more pathogenic variant of ZIKV Dakar (M. Gorman and M. Diamond, unpublished results). DENV-2 (strain D2S20) was obtained as a gift (S. Shresta, La Jolla, CA). Virus stocks were propagated in mycoplasma-free Vero cells and titrated by focus-forming assay (FFA), as described previously^3^.

### Mouse infection experiments

WT C57BL/6 mice were purchased commercially (Jackson Laboratories) and congenic *Rag1*^-/-^ mice were bred at Washington University in a pathogen-free facility. Congenic *Axl*^-/-^ mice were described previously^31^. Seven week-old mice were inoculated by subcutaneous route in the footpad with 10^3^ (H/PF/2013) or 10^6^ (Dakar 41519 or DENV-2) FFU in a volume of 50 μl. One-day prior to inoculation with virus, mice were treated with 0.5 or 2 mg of an Ifnar1-blocking mAb (MAR1-5A3) or isotype control mAb (GIR-208) by intraperitoneal injection^3^. At different days after infection, tissues were harvested and processed as described below. Testis and epididymis collected from infected male mice were processed for H & E staining, IF and confocal microscopy, ISH, and viral titer analysis as described previously^32^. Testis also were examined macroscopically and weighed. At days 14, 21, and ∼42 after ZIKV Dakar infection, the macroscopic damage as evidenced by reduction in size was often uniformly bilateral, although some asymmetry in testis (right versus left) size was observed. Randomization and blinding of the animal experiments were not performed, and samples sizes were not calculated a priori.

### CASA

Mature sperm from the caudal epididymis of uninfected or virus-infected mice were collected immediately after euthanasia as reported earlier^33^. The sperm suspension in vitrofert medium (Cook Medical) was analyzed for total sperm count by CASA using the HTM-IVOS Vs12 integrated visual optical system motility analyzer (Hamilton-Thorne Research, Beverly, MA) as previously described^34^. For studies at day ∼42 after infection, mice were harvested at day 41 (*n* = 3), 42 (*n* = 4), 43 (*n* = 3), and 48 (*n* =1) after infection with 10^3^ to 10^6^ FFU of ZIKV-Dakar. All measurements of total and motile sperm were made within 60 min of dissection of cauda epididymis.

### Testosterone and inhibin B levels

Total homogenates of testes from uninfected or ZIKV-infected mice were assayed for testosterone and inhibin B levels by radioimmunoassay as described^17^ using the Research in Reproduction Ligand Assay and Analysis Core at the University of Virginia.

### Fertility studies

Age-matched uninfected or ZIKV-infected WT C57BL/6 males (at days 7, 16 or 26 after infection, *n* = 4 to 5 at each time point) were mated with single 8 week-old female WT C57BL/6 mice. Five days later, males were removed from the cage to isolate the females. Ten days later, female mice (*n* = 14 to 15 for each group) were euthanized, evaluated for pregnancy, and the number of viable or resorbed fetuses was counted. Because sperm from mice can be obtained only at euthanasia, we were unable to perform longitudinal studies and correlate directly sperm counts after ZIKV infection with fertility rates.

### Viral burden

ZIKV- or DENV-infected mice were euthanized on specific days. Testes, epididymis, and other tissues were weighed and homogenized with zirconia beads in a MagNA Lyser instrument (Roche Life Science) in 200 μl of PBS. All homogenized tissues from infected animals were stored at −80°C. With some samples, viral burden was determined by plaque assay on Vero cells^35^. Sperm were subjected to three rapid freeze-thaw cycles to release infectious virus. Other samples were extracted with the RNeasy Mini Kit. ZIKV and DENV RNA levels were determined by one-step quantitative reverse transcriptase PCR (qRT-PCR) on an ABI 7500 Fast Instrument using standard cycling conditions. Viral burden was expressed on a log_10_ scale as viral RNA equivalents per g or ml after comparison with a standard curve produced using serial 10-fold dilutions of ZIKV or DENV RNA as described previously^35^. For ZIKV, the following primer sets were used: 1183F: 5′-CCACCAATGTTCTCTTGCAGACATATTG-3′; 1268R: 5′-TTCGGACAGCCGTTGTCCAACACAAG-3′; and probes (1213F): 5′-56-FAM/AGCCTACCTTGACAAGCAGTC/3IABkFQ-3′.

### Histology and immunohistochemistry

Tissues were harvested at necropsy and fixed overnight in 4% paraformaldehyde (PFA) in PBS. Subsequently, 5 μm-thick testis and epididymal sections from infected and uninfected mice were processed for histology by H & E staining. For immunohistochemistry, the tissue sections were incubated with mouse primary monoclonal anti-CD45 (610266; BD Biosciences), anti-ETV5 (ab102010; Abcam), and anti-GATA4 (ab84593; Abcam), rabbit polyclonal anti-Lin28a (3978S, Cell Signaling), rat polyclonal anti-TRA98 (ab82527; Abcam), rat polyclonal anti-F4/80 (ab6640; Abcam), or goat polyclonal anti-3β-HSD antibodies (SC-30820, Santa Cruz Biotechnology). After washing, slides were stained with Alexa Fluor 488- or, Alexa Fluor 546-conjugated goat anti-rabbit, goat anti-mouse, or donkey anti-goat (1:1000; A11008, A11081, A11030, or A11056; ThermoFisher Scientific) secondary antibodies for 1 h, and mounted with prolong gold anti-fade mount containing the nuclear counter stain, DAPI (ThermoFisher Scientific). Immunostaining was detected by confocal microscopy (Leica SPE100, Germany).

### Viral RNA *in situ* hybridization

RNA ISH was performed using RNAscope 2.5 (Advanced Cell Diagnostics) according to the manufacturer's instructions. PFA-fixed paraffin-embedded tissue sections were deparaffinized by incubating for 60 min at 60°C. Endogenous peroxidases were quenched with H_2_O_2_ for 10 min at room temperature. Slides were boiled for 15 min in RNAscope Target Retrieval Reagents and incubated for 30 min in RNAscope Protease Plus before probe hybridization. The probe targeting ZIKV RNA was designed and synthesized by Advanced Cell Diagnostics (Catalog #467871). Positive (targeting *plr2a* gene) and negative (targeting bacterial gene *dapB*) control probes also were obtained from Advanced Cell Diagnostics (Catalog #312471 and #310043, respectively). Tissues were counterstained with Gill's hematoxylin and visualized using bright-field microscopy.

### Data analysis

All data were analyzed with GraphPad Prism software. For viral burden analysis, the log_10_ transformed titers were analyzed by the Mann-Whitney test or a Kruskal-Wallis one-way ANOVA. A *P* value of < 0.05 indicated statistically significant differences.

### Data availability

The datasets generated during and/or analyzed during the current study are available from the corresponding author on reasonable request.

## Extended Data

**Extended Data Figure 1:**
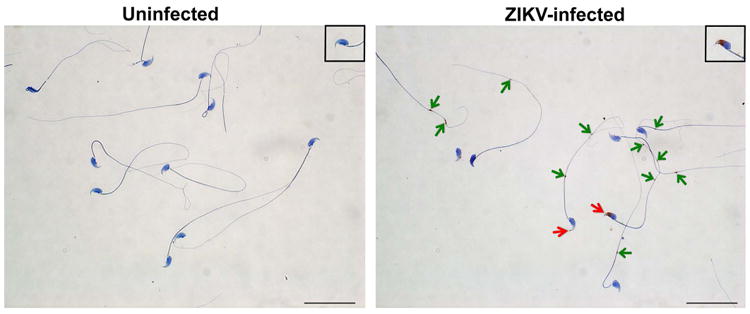
ZIKV infection of mature sperm Mature sperm was harvested from the caudal epididymis of uninfected (*left*) or ZIKV-infected (day 7, *right*) mice and processed by ISH with a ZIKV-specific probe. Staining for viral RNA is seen in the ZIKV-infected samples at the head (inset, *red arrow*) and in the cytoplasmic droplets (inset, *green arrows*) in the sperm flagellum. Scale bar = 50 μm. Staining was quantitated by microscopy: (i) Uninfected: 81 sperm counted, 0 positive for staining in head, 0 positive for staining in tail; (ii) ZIKV-infected: 93 sperm counted, 25 (27%) positive for staining in head, 57 (61%) positive for staining in tail.

**Extended Data Figure 2:**
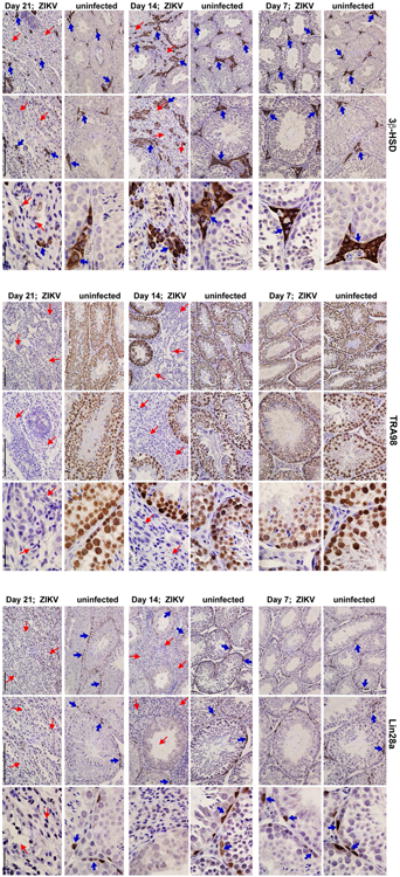
Temporal loss of cellularity in the testis after ZIKV infection Seven week-old WT C57BL/6 mice were treated with 0.5 mg of anti-Ifnar1 at day -1 prior to subcutaneous inoculation of mouse-adapted ZIKV Dakar. Immunohistochemical analysis was performed on testis tissues collected from uninfected (*top panels*) or ZIKV-infected animals (days 7, 14 or 21 after infection; *bottom panels*) at 20× (*left two images*) and 40× (*right image*) magnification. Staining was performed with antibodies against 3β-HSD (Leydig cells, *left panels*), TRA98 (germ cells, *middle panels*), and Lin28a (type A undifferentiated and type B spermatogonia, *right panels*). Blue arrows indicate staining of Leydig cells (*left panels*), germ cells (*middle panels*), and spermatogonial stem cells (*right panels*). Red arrows indicate areas of virus-induced damage and loss of tissue integrity and specific cellularity. Scale bars = 200, 200, and 50 μm for the grouping of the three sets of images.

**Extended Data Figure 3:**
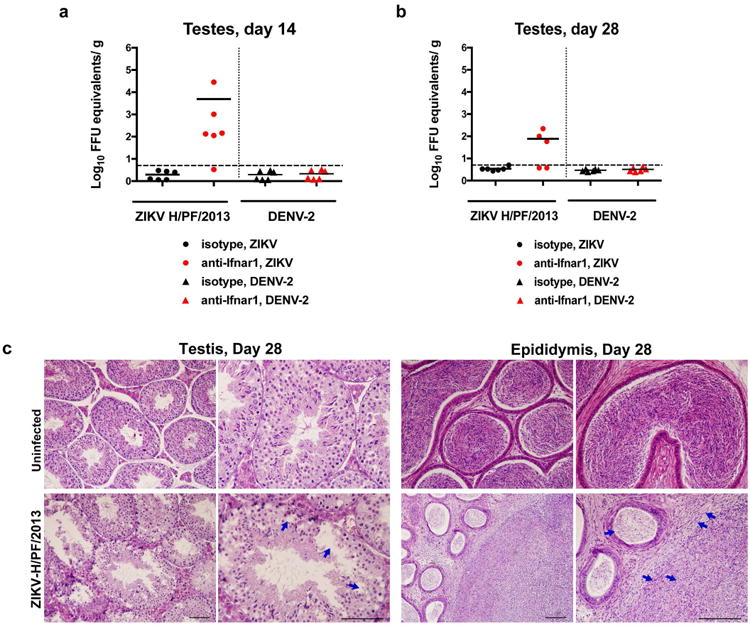
Histology of the testes at day 28 after infection with ZIKV H/PF/2013 Seven week-old WT C57BL/6 mice were treated with PBS or anti-Ifnar1 at day -1 prior to subcutaneous inoculation in the footpad with 10^3^ FFU of ZIKV H/PF/2013 or 10^6^ FFU of DENV-2. Testes were collected at day 14 (**a**) or 28 (**b**) after infection and analyzed for viral RNA by qRT-PCR. Results are pooled from two independent biological experiments and each symbol represents data from an individual mouse. Bars indicate mean values. **c**. Histological analysis of PFA-fixed testis (*left panels*) and epididymis (*right panels*) tissues collected from uninfected or ZIKV-infected animals at day 28 at 20× (*left*) and 40× (*right*) magnification. Arrows indicate loss of germ cells and vacuoles in the testis (red), involution of epididymal lumens (yellow) with a mass of residual sperm (blue) and thickened epithelium (green). The images are representative of several independent experiments. Scale bars are indicated in the bottom right corner of the panels. Scale bars = 200 μm.

**Extended Data Figure 4:**
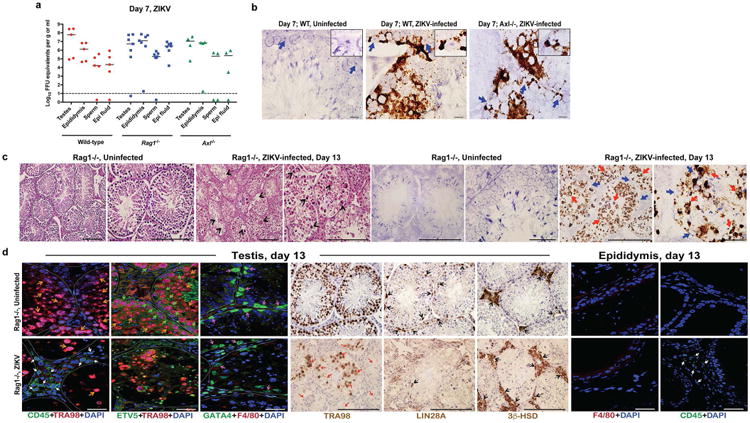
ZIKV infection of the testis and epididymis at day 7 in *Axl*^-/-^ and *Rag1*^-/-^ mice Seven week-old WT, *Axl*^-/-^, or *Rag1*^-/-^ C57BL/6 mice were treated with 0.5 mg of anti-Ifnar1 at day -1 prior to subcutaneous inoculation in the footpad with 10^6^ FFU of mouse-adapted ZIKV Dakar. **a**. The indicated tissues were collected at day 7 after infection and analyzed for viral RNA by qRT-PCR. Dashed lines indicate limit of detection of the assays. **b**. ISH of testis from uninfected or ZIKV-infected WT and *Axl*^-/-^ mice at day 7 with a ZIKV-specific probe. Dark blue arrows indicate Sertoli cells. (*Inset*) In sections from infected WT and *Axl*^-/-^mice, the cytoplasm of Sertoli cells is positive for ZIKV RNA (dark brown) with signal absent from prominent nuclei and nucleoli. Scale bar = 50 μm. **c**. Histology (H & E, *left two panels*) and ISH (*right two panels*) of testis from age-matched uninfected or ZIKV-infected (day 13) *Rag1*^-/-^ mice at 20× (*left*) and 40× (*right*) magnification for each pair. Scale bars = 200 and 50 μm. In H & E stained testis sections arrows indicate loss of germ cells, and presence of multi-nucleated giant and necrotic cells from ZIKV-infected *Rag1*^-/-^ mice. In ISH, red and blue arrows indicate distribution of ZIKV RNA and Sertoli cells, respectively. **d**. IF (*three left and two right panels*) and IHC (*three middle*) staining of uninfected or ZIKV-infected (day 13) testis and epididymis from *Rag1*^-/-^ mice with antibodies to CD45, TRA98, ETV5, GATA4, Lin28a, 3b-HSD, F4/80 as described in Figure 1 and Extended Data Figure 2. Colored arrows indicate staining for leukocytes (CD45, white), germ cells (TRA98, orange), Sertoli cells (GATA4, magenta), BTB (ETV5, green), type A undifferentiated and type B spermatogonia (Lin28a, black), and Leydig cells (3β-HSD, black) in respective panels. In the IHC staining panels with TRA98, red arrows indicate dying or dead germ cells and tubules without germ cells. White lines demarcate tubules in the seminiferous epithelium. Scale bars = 200 μm for IHC and 50 μm for IF. The images are representative from several different animals.

**Extended Data Figure 5:**
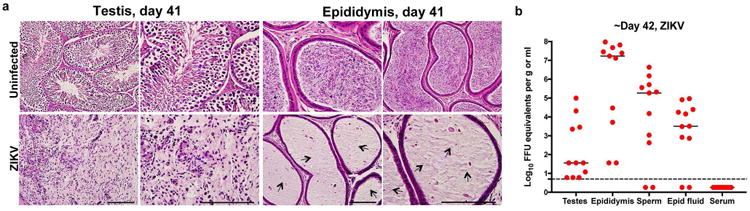
ZIKV infection of the testis and epididymis at ∼day 42. a–b Seven week-old WT C57BL/6 mice were treated with anti-Ifnar1 at day -1 prior to subcutaneous inoculation in the footpad with 10^5^ FFU of mouse-adapted ZIKV Dakar. **a**. Testis (*left panels*) and epididymis (*right panels*) were collected at day 41 after infection or from age-matched uninfected mice, fixed with PFA, sectioned, stained with H & E, and imaged at 20× (*left*) and 40× (*right*) magnification. Arrows show epididymal lumen void of sperm. The images are representative of sections from several independent animals. Scale bars are indicated in the bottom right corner of the panels. Scale bars = 200 and 50 μm. **b**. The indicated tissues and cells were collected at ∼day 42 after infection (days 41 (*n* = 3), 42 (*n* = 4), 43 (*n* = 3), and 48 (*n* =1)) and analyzed for viral RNA by qRT-PCR. Dashed line indicates the limit of detection of the assay. Results are pooled from two to three independent biological experiments and each symbol represents data from an individual mouse. Bars indicate median values.

## Figures and Tables

**Figure 1:**
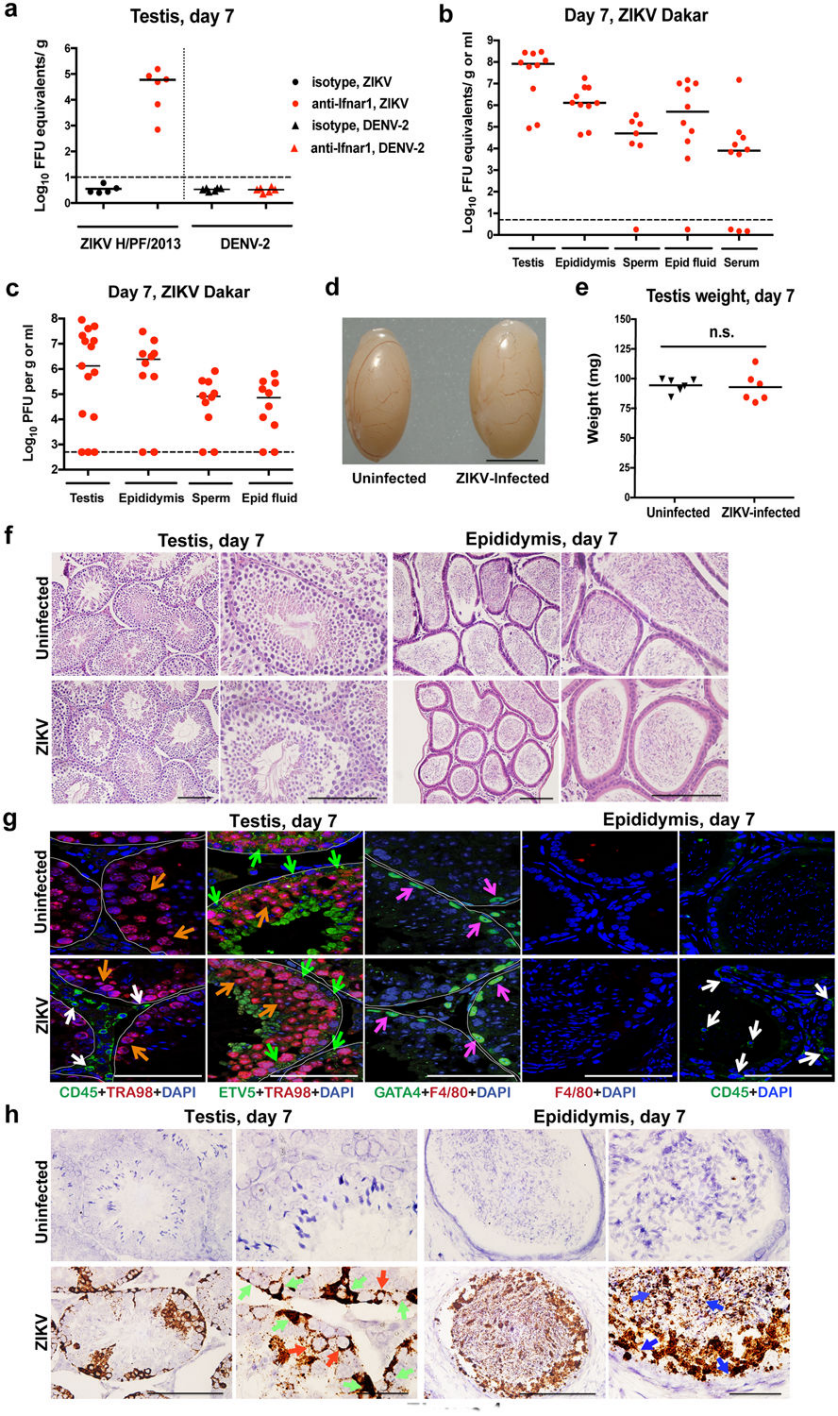
ZIKV infection of the testis and epididymis at day 7. a–c Seven week-old WT mice were treated with an isotype control (**a**) or anti-Ifnar1 mAb (2 mg (**a**) or 0.5 mg (**b–c**)) at day -1 prior to subcutaneous inoculation with 10^3^ FFU of ZIKV H/PF/2013 (**a**), 10^6^ FFU of DENV-2 (**a**), or mouse-adapted ZIKV Dakar (**b–c**). Tissues and cells were collected at day 7 after infection and analyzed for viral RNA by qRT-PCR (**a–b**) or infectious virus by plaque assay (**c**). Dashed lines indicate limit of detection. Results are pooled from two to three independent experiments and each symbol represents data from an individual mouse. Bars indicate median values. Viral RNA was normalized to a standard curve from RNA isolated from infectious virus. **d** Representative image of testis from an uninfected and ZIKV Dakar-infected mouse at day 7; scale bar = 2 mm. **e**. Weight of testis from uninfected and ZIKV-infected mice at day 7. Results are pooled from two independent experiments. Mean values were not statistically different (n.s.; unpaired t test). **f-h.** Histological, immunohistochemical, and ISH analysis of testis (*left panels*) and epididymis (*right panels*) collected from uninfected or ZIKV-infected animals at 20× (*left*) and 40× (*right*) magnification. f. Hematoxylin and eosin (H & E) staining. **g.** Immunofluorescence (IF) staining of uninfected or ZIKV-infected testis and epididymis tissue sections with antibodies to CD45 (pan-leukocyte), TRA98 (germ cells), ETV5 (BTB), GATA4 (Sertoli cells), and F4/80 (macrophages). Arrows indicate staining for leukocytes (white), germ cells (orange), Sertoli cells (magenta), and BTB (green). White lines demarcate tubules of seminiferous epithelium. **h**. ISH with a ZIKV-specific probe. Arrows indicate cells positive for ZIKV RNA (spermatogonia and primary spermatocytes (red), Sertoli cells (green), and epididymis lumenal sperm (blue)). The images in panels **f-h** are representative of several independent experiments. Scale bars = 200 and 50 μm for H & E and ISH and 50 μm for IF.

**Figure 2:**
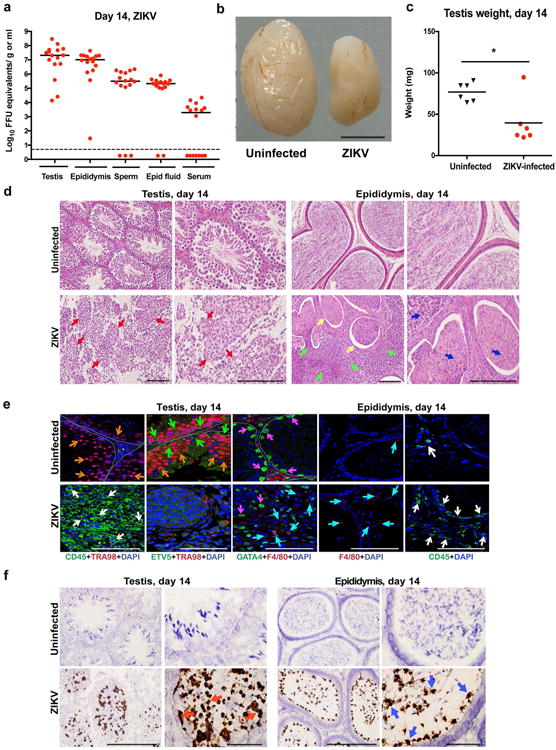
ZIKV infection of the testis and epididymis at day 14. a–b Seven week-old WT mice were treated with 0.5 mg of anti-Ifnar1 at day -1 prior to subcutaneous inoculation of mouse-adapted ZIKV Dakar. Tissues and cells were collected at day 14 and analyzed for viral RNA by qRT-PCR (**a**). Dashed lines indicate limit of detection. Results are pooled from three independent experiments. Bars indicate median values. **b**. Representative images of testis from an uninfected and ZIKV-infected mouse at day 14; scale bar = 2 mm. **c**. Weight of testis from uninfected and ZIKV-infected mice at day 14. Results are pooled from two independent experiments (*, *P* < 0.05, Mann-Whitney test). **d–f**. Histological, immunohistochemical, and ISH analysis of testis (*left panels*) and epididymis (*right panels*) tissues collected from uninfected or ZIKV-infected animals at 20× (*left*) and 40× (*right*) magnification. **d**. H & E staining. Arrows denote involution of seminiferous tubules in the testis (red), constricted epididymal lumens (yellow) with a mass of residual sperm (blue) and thickened epithelium (green). **e**. IF staining of uninfected or ZIKV-infected testes and epididymis tissues as described in Figure 1. Arrows indicate staining for leukocytes (white), germ cells (orange), Sertoli cells (magenta), BTB (green), and macrophages (cyan). White lines demarcate tubules in the seminiferous epithelium. **f**. ISH. Arrows indicate cells positive for ZIKV RNA (testicular cells (red) and epididymis lumenal sperm and cilia on the inner layer of epididymal epithelium (blue)). The images in panels **d–f** are representative of several independent experiments. Scale bars = 200 and 50 μm for H & E and ISH and 50 μm for IF.

**Figure 3:**
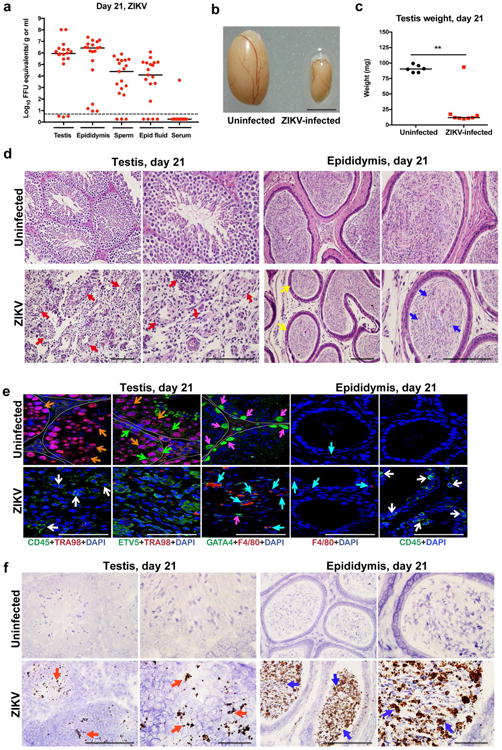
ZIKV infection of the testis and epididymis at day 21 **a** Seven week-old WT mice were treated with 0.5 mg of anti-Ifnar1 at day -1 prior to subcutaneous inoculation of mouse-adapted ZIKV Dakar. Tissues and cells were collected at day 21 after infection and analyzed for viral RNA by qRT-PCR. Dashed lines indicate limit of detection. Results are pooled from two independent experiments. Bars indicate median values. **b**. Representative images of testis from an uninfected and ZIKV-infected mouse at day 21; scale bar = 2 mm. **c**. Weight of testis from uninfected and ZIKV-infected mice at day 21. Results are pooled from two independent experiments (*, *P* < 0.05, Mann-Whitney test). **d**. Histological analysis of testis (*left panels*) and epididymis (*right panels*) tissues collected from uninfected or ZIKV-infected animals at 20× (*left*) and 40× (*right*) magnification and stained with H & E. Arrows indicate involution of seminiferous tubules in the testis (red), shrunken epididymal lumens (yellow) with a mass of residual sperm (blue). **e**. IF staining of uninfected or ZIKV-infected testis and epididymis tissues as described in Figure 1 and 2. Arrows indicate staining for leukocytes (white), germ cells (orange), Sertoli cells (magenta), BTB (green), and macrophages (cyan). White lines demarcate tubules in the seminiferous epithelium **f**. ISH. Arrows indicate cells positive for ZIKV RNA (testicular cells (red) and epididymal lumenal sperm (blue)). The images in panels **d-f** are representative of several independent experiments. Scale bars = 200 and 50 μm for H & E and ISH and 50 μm for IF.

**Figure 4:**
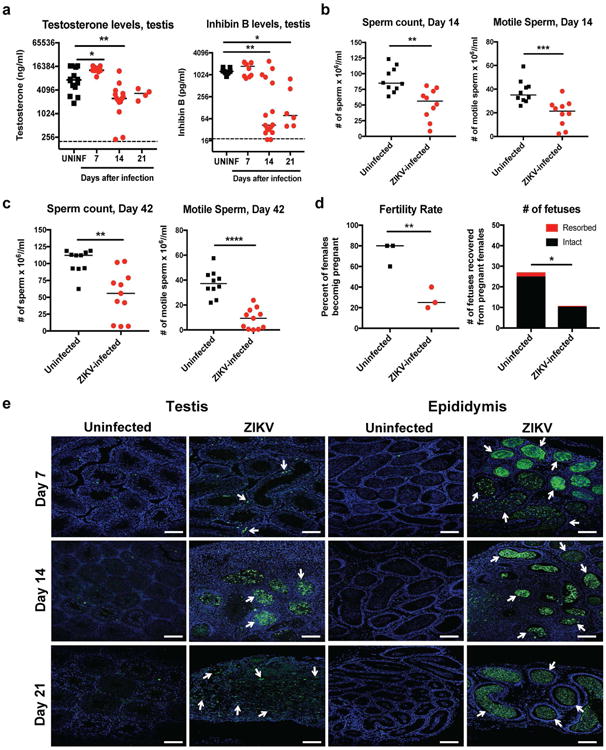
Consequences of ZIKV infection of the testis and epididymis **a** Testosterone (*left*) and inhibin B (*right*) levels of testis homogenates from uninfected (UNINF) and ZIKV-infected (days 7, 14, or 21) mice. **b–c**. Computer-assisted sperm analysis (total (*left panels*) and motile (*right panels*)) on samples obtained from the caudal epididymis of ZIKV-infected males immediately after euthanasia at days (**b**) 14 or (**c**) ∼42 (range of 41 to 48 days) after infection or age-matched uninfected males. **a–c**. Results are pooled from two to five independent experiments. Bars indicate median values, and differences between uninfected and ZIKV-infected animals were evaluated (**a**, *, *P* < 0.05; **, *P* < 0.01; ***, *P* < 0.001; ANOVA (Kruskal-Wallis) with a multiple comparison correction; **b–c**, **, *P* < 0.01; ***, *P* < 0.001; ****, *P* < 0.0001; Mann-Whitney test. Dashed lines indicate the limit of sensitivity of the assay. d. Fertility studies. Age-matched uninfected or ZIKV-infected males (at days 7, 16 or 26 after infection (*n* = 4 to 5 male mice for each time point)) were mated with individual 8 week-old female WT mice (*n* = 4-5 female per round with 3 independent rounds performed) for five days and then separated. Ten days later, we evaluated the pregnancy rate (*left,* symbols correspond to the percentage of 5 females becoming pregnant for a given trial) and the total number of viable or resorbed fetuses for each round (*right*) (*, *P* < 0.05; **, *P* < 0.01; unpaired Student's t test). **e**. TUNEL staining of testis (*left panels*) and epididymis (*right panels*) from uninfected or ZIKV-infected mice at days 7, 14, or 21. TUNEL staining in germ and somatic cells of the testis and lumenal sperm in the epididymis is shown (green staining and white arrows). The images are representative of several independent experiments. Scale bars = 100 μm.

## References

[1] Coyne CB, Lazear HM (2016). Zika virus - reigniting the TORCH. Nat Rev Microbiol.

[2] Matheron S (2016). Long Lasting Persistence of Zika Virus in Semen. Clin Infect Dis.

[3] Lazear HM (2016). A Mouse Model of Zika Virus Pathogenesis. Cell Host Microbe.

[4] Rossi SL (2016). Characterization of a Novel Murine Model to Study Zika Virus. Am J Trop Med Hyg.

[5] Foy BD (2011). Probable non-vector-borne transmission of Zika virus, Colorado, USA. Emerg Infect Dis.

[6] Musso D (2015). Potential sexual transmission of Zika virus. Emerg Infect Dis.

[7] Deckard DT (2016). Male-to-Male Sexual Transmission of Zika Virus - Texas. MMWR Morb Mortal Wkly Rep.

[8] Grant A (2016). Zika Virus Targets Human STAT2 to Inhibit Type I Interferon Signaling. Cell Host Microbe.

[9] Morrow CM (2007). ETV5 is required for continuous spermatogenesis in adult mice and may mediate blood testes barrier function and testicular immune privilege. Ann N Y Acad Sci.

[10] Mullen TE, Kiessling RL, Kiessling AA (2003). Tissue-specific populations of leukocytes in semen-producing organs of the normal, hemicastrated, and vasectomized mouse. AIDS Res Hum Retroviruses.

[11] Lu Q (1999). Tyro-3 family receptors are essential regulators of mammalian spermatogenesis. Nature.

[12] Savidis G (2016). Identification of Zika Virus and Dengue Virus Dependency Factors using Functional Genomics. Cell Rep.

[13] Nowakowski TJ (2016). Expression Analysis Highlights AXL as a Candidate Zika Virus Entry Receptor in Neural Stem Cells. Cell Stem Cell.

[14] Hamel R (2015). Biology of Zika Virus Infection in Human Skin Cells. J Virol.

[15] Tabata T (2016). Zika Virus Targets Different Primary Human Placental Cells, Suggesting Two Routes for Vertical Transmission. Cell Host Microbe.

[16] Liu S, DeLalio LJ, Isakson BE, Wang TT (2016). AXL-Mediated Productive Infection of Human Endothelial Cells by Zika Virus. Circulation research.

[17] Dierich A (1998). Impairing follicle-stimulating hormone (FSH) signaling in vivo: targeted disruption of the FSH receptor leads to aberrant gametogenesis and hormonal imbalance. Proc Natl Acad Sci U S A.

[18] Wu H (2016). Mumps virus-induced innate immune responses in mouse Sertoli and Leydig cells. Sci Rep.

[19] Le Goffic R (2003). Mumps virus decreases testosterone production and gamma interferon-induced protein 10 secretion by human leydig cells. J Virol.

[20] Oehler E (2014). Zika virus infection complicated by Guillain-Barre syndrome--case report, French Polynesia, December 2013. Euro Surveill.

[21] Carteaux G (2016). Zika Virus Associated with Meningoencephalitis. N Engl J Med.

[22] Brasil P (2016). Zika Virus Infection in Pregnant Women in Rio de Janeiro - Preliminary Report. N Engl J Med.

[23] Mansuy JM (2016). Zika virus in semen of a patient returning from a non-epidemic area. Lancet Infect Dis.

[24] Turmel JM (2016). Late sexual transmission of Zika virus related to persistence in the semen. Lancet.

[25] D'Ortenzio E (2016). Evidence of Sexual Transmission of Zika Virus. N Engl J Med.

[26] Zhao H (2016). Structural Basis of Zika Virus-Specific Antibody Protection. Cell.

[27] Barrows NJ (2016). A Screen of FDA-Approved Drugs for Inhibitors of Zika Virus Infection. Cell Host Microbe.

[28] Larocca RA (2016). Vaccine protection against Zika virus from Brazil. Nature.

[29] Torres JR, Martinez N, Moros Z (2016). Microhematospermia in acute Zika virus infection. Int J Infect Dis.

[30] Mansuy JM (2016). Zika virus in semen and spermatozoa. Lancet Infect Dis.

